# Association between lncRNA‐*H19* polymorphisms and hepatoblastoma risk in an ethic Chinese population

**DOI:** 10.1111/jcmm.16124

**Published:** 2020-11-24

**Authors:** Tianbao Tan, Jiahao Li, Yang Wen, Yan Zou, Jiliang Yang, Jing Pan, Chao Hu, Yuxiao Yao, Jiao Zhang, Yijuan Xin, Suhong Li, Huimin Xia, Jing He, Tianyou Yang

**Affiliations:** ^1^ Department of Pediatric Surgery Guangzhou Women and Children’s Medical Center Guangzhou Medical University Guangzhou China; ^2^ First Affiliated Hospital of Sun Yat‐Sen University Guangzhou China; ^3^ Department of Pediatric Surgery First Affiliated Hospital of Zhengzhou University Zhengzhou China; ^4^ Clinical Laboratory Medicine Center of PLA Xijing Hospital Air Force Medical University Xi'an, Shaanxi China; ^5^ Department of Pathology Children's Hospital and Women's Health Center of Shanxi Taiyuan China

**Keywords:** genetic variant, H19, hepatoblastoma, lncRNA

## Abstract

*H19* polymorphisms are associated with increased susceptibility to several cancers; however, their role in hepatoblastoma remains unclear. In this study, we investigated the association between three *H19* polymorphisms (rs2839698 G>A, rs3024270 C>G, rs217727 G>A) and hepatoblastoma susceptibility in 213 hepatoblastoma patients. The rs2839698 and rs3024270 polymorphisms were associated with significantly increased hepatoblastoma risk, with the GG genotype associated with a higher risk of hepatoblastoma than the CC genotype at the rs3024270 locus. The rs217727 polymorphism was associated with significantly decreased hepatoblastoma risk, with the AG genotype associated with a lower risk of hepatoblastoma than the GG genotype. These findings were confirmed by combined analysis, and stratification analysis revealed that age, gender and clinical stage were associated with increased hepatoblastoma susceptibility. The GGG and AGG haplotypes were significantly associated with increased hepatoblastoma risk compared with the GCA reference (rs2839698, rs3024270, rs217727). The rs2839698 and rs3024270 polymorphisms correlated with decreased MRPL23‐AS1 expression, whereas the rs217727 polymorphism was associated with increased MRPL23‐AS1 expression. Overall, the *H19* rs2839698, rs3024270 and rs217727 polymorphisms were associated with hepatoblastoma susceptibility in a Chinese Han population.

## 1 INTRODUCTION

Hepatoblastoma is the most common hepatic malignancy in children, occurring in around 1/1 000 000 individuals and accounting for over 90% of primary hepatic malignancies in children <5 years old.^1^ Hepatoblastoma usually presented with a large abdominal mass and increased alpha‐fetoprotein (AFP) value.^2^ Most hepatoblastomas are sporadic, but some are related to genetic aberrations and had a family history of cancers.^3^ The aetiology and pathogenesis of hepatoblastoma remain largely unknown. However, increasing evidence suggested that chromosomal abnormalities, genetic aberrations and various adverse factors during pregnancy may contribute to the development of hepatoblastoma.^4^ The management of hepatoblastoma is multidisciplinary, consisting of surgery, chemotherapy and liver transplantation, and the 5‐year overall survival rate is approximately 80%.^5^ Nonetheless, the treatment of high‐risk hepatoblastomas remains challenging.

Hepatoblastoma is associated with several genetic syndromes, such as Edwards syndrome, Beckwith‐Wiedemann syndrome (BWS) and familial adenomatous polyposis syndrome.^4^, ^6^ Evidence suggests that children with BWS have a much higher risk of developing hepatoblastoma than those without.^6^ The abnormal methylation of tumour‐specific genes in differentially methylated regions (DMRs), such as 11p15.5 and 20q13.3, has been observed prior to hepatoblastoma development in patients with BWS.^6^, ^7^ In addition, the DNA hypermethylation of IGF2 biallelic expression has been observed in hepatoblastoma.^8^ Indeed, abnormal DNA methylation in imprinted DMRs is thought to be a key mechanism in the malignant transformation of progenitor cells in various tissues, including the liver.^9^ Additionally, previous studies have shown that the classical Wnt signalling pathway, which is involved in somatic and germline mutations in various genes, is commonly deregulated in hepatoblastoma.^10^

*H19* is a 3.0 kb gene located on human chromosome 11, and its transcript is a highly conserved long non‐coding RNA (lncRNA), known as lncRNA‐H19.^11^ H19 plays an important role in tumorigenesis via transcriptional regulation, post‐transcriptional regulation and epigenetic regulation. Moreover, H19 is associated with the occurrence of various malignant tumours, such as breast cancer, bladder cancer and gastric cancer.^12^ H19 has been shown to promote cell cycle progression in breast cancer, with its deletion arresting breast cancer cells in the pre‐S‐phase of the cell cycle.^13^ Moreover, knockdown of H19 inhibits the growth of hepatocellular carcinoma (HCC) and gastric cancer cells under hypoxia recovery conditions,^14^ whereas H19 overexpression partially suppresses p53 activation in gastric cancer cells.^15^ In addition, Zhu et al^16^ found that silencing H19 can inhibit the proliferation of ovarian cancer SKOV3 cells and is related to the regulation of certain cell cycle‐ and apoptosis‐related proteins, thus exerts an inhibitory effect on ovarian cancer cell growth. A study by Zhang et al found that high H19 expression positively correlates with the lymph node metastasis of cancer cells and tumour size, and can be used as an independent prognostic factor for the overall survival of non‐small‐cell lung cancer (NSCLC). Furthermore, the proto‐oncogene c‐myc can directly regulate H19, with *H19* gene silencing significantly inhibiting the proliferation of NSCLC cells.^17^ Taken together, these studies indicate that H19 is closely related to the occurrence and development of tumours; however, its role may differ in different types of cancer. For instance, H19 can exhibit proto‐oncogene activities that promote tumour development and growth,^18^ but can act as a tumour suppressor gene in other tumours by inhibiting tumour proliferation, metastasis and invasion.^19^, ^20^

Recent evidence has shown that polymorphic sites in the *H19* gene are associated with susceptibility to a variety of malignancies.^21^ Lin et al^22^ found that rs217727 C>T in *H19* may be associated with an increased risk of breast cancer, whereas Xia et al^23^ found no significant association between rs3741219 polymorphisms and breast cancer susceptibility in a Chinese population. Others have found that the *H19* rs3741219 polymorphism is associated with increased HCC risk ^24^ and that the rs2839698 polymorphism is associated with an increased risk of various gastrointestinal cancers, with this association being even more prominent in the Asian population.^25^ Despite increasing evidence suggesting that *H19* polymorphisms are associated with increased susceptibility to many cancers, the association between *H19* polymorphisms and hepatoblastoma susceptibility has not yet been investigated. Therefore, we investigated the association between three *H19* polymorphisms (rs2839698 G>A, rs3024270 C>G, rs217727 G>A) and hepatoblastoma susceptibility, using the odds ratio and 95% confidence interval to determine the strength of the association.

## 2 MATERIALS AND METHODS

### 2.1 Study population

We enrolled 213 patients with hepatoblastoma and 958 cancer‐free controls in this study.^26^, ^27^, ^28^ The initial diagnosis of hepatoblastoma was made based upon ultrasound detected liver mass and elevated AFP value. Then, tumour tissue was obtained by image‐guided liver biopsy. All cases of hepatoblastoma were diagnosed by pathological examination, and no direct blood relationship was found among any of the cases. Patients older than 18 years or with HCC were excluded. Healthy controls were recruited in hospitals. All healthy controls had no history of malignancy and were matched to the hepatoblastoma cases by age (±5 years), gender, ethnicity and geographical region (as shown in Table S1). This study was approved by the Institutional Review Board of Guangzhou Women and Children's Medical Center (approval number 2017120101), with written informed consent provided by the parents or legal guardians of the participants.

### 2.2 SNP selection and genotyping

We selected *H19* gene SNPs of potential functional interest from the dbSNP database (http://www.ncbi.nlm.nih.gov/) and SNPinfo (http://snpinfo.niehs.nih.gov/) for analysis (rs2839698 G>A, rs3024270 C>G and rs217727 G>A). Total genomic DNA was isolated from the peripheral blood leucocytes of each patient using a TIANamp Blood DNA Kit (TianGen Biotech Co., Ltd.). *H19* gene SNPs were genotyped by TaqMan real‐time PCR,^26^, ^27^, ^28^ with eight blank wells in each 384‐well plate containing water as negative controls. We also randomly repeated the genotyping of 10% of the samples, achieving a 100% concordance rate.

### 2.3 Statistical analysis

To test for HWE in the controls, we used the observed genotype frequencies. A two‐sided chi‐squared test was used to compare the allele frequencies and demographic variables of two groups. Associations between genotypes and hepatoblastoma risk were evaluated using the OR and 95% CI calculated by logistic regression analysis. Statistical analyses were performed using SAS software version 9.4 (SAS Institute). *P*‐values of <.05 were considered significant.

## 3 RESULTS

### 3.1 Associations between H19 polymorphisms and hepatoblastoma susceptibility

In this study, we genotyped the frequencies of three *H19* single‐nucleotide polymorphisms (SNPs) in 213 patients with hepatoblastoma and 957 controls, as shown in Table 1. The genotype frequencies of all three selected SNPs were in Hardy‐Weinberg equilibrium (HWE; rs2839698, *P* = .568; rs3024270, *P* = .422; rs217727, *P* = .381). At the *H19 rs2839698* locus, the proportion of GG, heterozygous AG, and mutated homozygous AA genotypes were 47.89, 36.62, and 15.49% in the case group and 45.87, 44.31, and 9.82% in the healthy control group, respectively. Compared to the GG genotype, there was no significant difference in the frequency of the AG and AA genotypes between the case and control groups (all *P* > .05). Moreover, *H19* rs2839698 polymorphism was associated with significantly increased risk of hepatoblastoma (recessive model: adjusted odds ratio (OR) = 1.68, 95% confidence interval (CI) = 1.10‐2.58, *P* = .017). At the *H19* rs3024270 locus, the CC, mutant heterozygous CG, and mutant homozygous GG genotypes were present at 23.47, 40.85, and 35.68% in the case group and 27.59, 51.10, and 21.32% in the healthy control group, respectively. The GG genotype increased the risk of hepatoblastoma compared to CC genotype (adjusted OR = 1.97, 95% CI = 1.32‐2.94, *P* = .0009). Moreover, rs3024270 polymorphism was also significantly associated with increased hepatoblastoma risk (recessive model: adjusted OR = 2.05, 95% CI = 1.49‐2.82, *P* < .0001). At the *H19* rs217727 locus, the GG, mutant heterozygous AG, and mutant homozygous AA genotypes were found at 59.15, 31.92, and 8.92% in the case group and 45.77, 42.84, and 11.39% in the healthy control group, respectively. The AG genotype reduced the risk of hepatoblastoma compared to the GG genotype (adjusted OR = 0.58, 95% CI = 0.42‐0.80, *P* = .0009), while H19 rs217727 polymorphism was associated with significantly lower hepatoblastoma risk (dominant model: adjusted OR = 0.58, 95% CI = 0.43‐0.79, *P* = .0004).

**Table 1:** Correlation between *H19* polymorphisms and hepatoblastoma susceptibility

Genotype	Cases (n = 213)	Controls (n = 957)	*P* ^a^	Crude OR (95% CI)	*P*	Adjusted OR (95% CI)^b^	*P* ^b^
rs2839698 G>A (HWE = 0.568)							
GG	102 (47.89)	439 (45.87)		1.00		1.00	
AG	78 (36.62)	424 (44.31)		0.79 (0.57‐1.09)	.157	0.79 (0.57‐1.09)	.156
AA	33 (15.49)	94 (9.82)		1.51 (0.96‐2.37)	.073	1.51 (0.96‐2.37)	.073
Additive			.470	1.09 (0.87‐1.35)	.470	1.08 (0.87‐1.35)	.471
Dominant	111 (52.11)	518 (54.13)	.594	0.92 (0.69‐1.24)	.594	0.92 (0.68‐1.24)	.591
Recessive	180 (84.51)	863 (90.18)	.016	**1.68 (1.10‐2.58)**	**.017**	**1.68 (1.10‐2.58)**	**.017**
rs3024270 C>G (HWE = 0.422)							
CC	50 (23.47)	264 (27.59)		1.00		1.00	
CG	87 (40.85)	489 (51.10)		0.94 (0.64‐1.37)	.746	0.94 (0.64‐1.37)	.748
GG	76 (35.68)	204 (21.32)		**1.97 (1.32‐2.94)**	**.0009**	**1.97 (1.32‐2.94)**	**.0009**
Additive			.0006	**1.44 (1.17‐1.78)**	**.0007**	**1.44 (1.17‐1.79)**	**.0007**
Dominant	163 (76.53)	693 (72.41)	.221	1.24 (0.88‐1.76)	.221	1.24 (0.88‐1.76)	.221
Recessive	137 (64.32)	753 (78.68)	<.0001	**2.05 (1.49‐2.82)**	**<.0001**	**2.05 (1.49‐2.82)**	**<.0001**
rs217727 G>A (HWE = 0.381)							
GG	126 (59.15)	438 (45.77)		1.00		1.00	
AG	68 (31.92)	410 (42.84)		**0.58 (0.42‐0.80)**	**.0009**	**0.58 (0.42‐0.80)**	**.0009**
AA	19 (8.92)	109 (11.39)		0.61 (0.36‐1.03)	.062	0.61 (0.36‐1.02)	.061
Additive			.002	**0.69 (0.55‐0.87)**	**.002**	**0.69 (0.55‐0.87)**	**.002**
Dominant	87 (40.85)	519 (54.23)	.0004	**0.58 (0.43‐0.79)**	**.0004**	**0.58 (0.43‐0.79)**	**.0004**
Recessive	194 (91.08)	848 (88.61)	.296	0.76 (0.46‐1.27)	.298	0.76 (0.46‐1.27)	.297
Combined effect of risk genotypes^c^							
0	86 (40.38)	518 (54.13)		1.00		1.00	
1	52 (24.41)	235 (24.56)		1.33 (0.91‐1.94)	.136	1.33 (0.91‐1.95)	.135
2	42 (19.72)	111 (11.60)		**2.28 (1.49‐3.48)**	**.0001**	**2.28 (1.50‐3.48)**	**.0001**
3	33 (15.49)	93 (9.72)		**2.14 (1.35‐3.38)**	**.001**	**2.14 (1.35‐3.38)**	**.001**
1‐3	127 (59.62)	439 (45.87)	.0003	**1.74 (1.29‐2.36)**	**.0003**	**1.74 (1.29‐2.36)**	**.0003**

Abbreviations: CI, confidence interval; HWE, Hardy‐Weinberg equilibrium; OR, odds ratio.

^a^ Chi‐squared test of genotype distribution differences between hepatoblastoma patients and cancer‐free controls.

^b^ Adjusted for age and gender.

^c^ Risk genotypes were carriers with rs2839698 AA, rs3024270 GG and rs217727 GG genotypes.

Combined analysis revealed that hepatoblastoma risk significantly increased as the number of risk alleles increased, with rs2839698 AA, rs3024270 GG and rs217727 GG considered risk alleles based on the primary effect of the individual locus. Subjects carrying two risk alleles (adjusted OR = 2.28, 95% CI = 1.50‐3.48, *P* = .0001), three risk alleles (adjusted OR = 2.14, 95% CI = 1.35‐3.38, *P* = .001) or one‐three risk alleles (adjusted OR = 1.74, 95% CI = 1.29‐2.36, *P* = .0003) all displayed a higher risk of hepatoblastoma than the group with no risk alleles.

### 3.2 Stratified analysis of H19 polymorphisms and hepatoblastoma risk

To further analyse the effects of the rs2839698, rs3024270 and rs217727 genotypes on hepatoblastoma susceptibility, we conducted stratification analyses based on age (<17 months, ≥17 months), gender and clinical stage (I + II, III + IV). As shown in Table 2, significant associations were detected in the following subgroups: children < 17 months (adjusted OR = 1.88; 95% CI = 1.23‐2.85, *P* = .003), children ≥ 17 months (adjusted OR = 1.62; 95% CI = 1.05‐2.51, *P* = .031), females (adjusted OR = 2.13; 95% CI = 1.37‐2.3.63, *P* = .001), males (adjusted OR = 1.47; 95% CI = 1.00‐2.17, *P* = .049), patients with clinical stage I + II tumours (adjusted OR = 2.00, 95% CI = 1.30‐3.08, *P* = .002) and patients with clinical stage III + IV tumours (adjusted OR = 1.92, 95% CI = 1.10‐3.37, *P* = .022).

**Table 2:** Stratification analysis of association between *H19* genotypes and hepatoblastoma susceptibility

Variables	rs2839698 (case/control)		AOR (95% CI)^a^	*P* ^a^	rs3024270 (case/control)		AOR (95% CI)^a^	*P* ^a^	rs217727 (case/control)		AOR (95% CI)^a^	*P* ^a^	Risk genotypes (case/control)		AOR (95% CI)^a^	*P* ^a^
	GG/AG	AA			CC/CG	GG			GG	AG/AA			0	1‐3		
Age, mo																
<17	97/408	17/46	1.55 (0.85‐2.82)	.155	73/354	41/100	**1.98 (1.27‐3.09)**	**.003**	68/203	46/251	**0.55 (0.36‐0.83)**	**.005**	45/250	69/204	**1.88 (1.23‐2.85)**	**.003**
≥17	83/455	16/48	1.83 (0.99‐3.37)	.054	64/399	35/104	**2.10 (1.32‐3.34)**	**.002**	58/235	41/268	**0.62 (0.40‐0.96)**	**.031**	41/268	58/235	**1.62 (1.05‐2.51)**	**.031**
Gender																
Female	69/346	15/33	**2.32 (1.19‐4.52)**	**.013**	54/304	30/75	**2.28 (1.36‐3.81)**	**.002**	51/159	33/220	**0.47 (0.29‐0.76)**	**.002**	32/220	52/159	**2.13 (1.37‐3.63)**	**.001**
Male	111/517	18/61	1.37 (0.78‐2.42)	0.270	83/449	46/129	**1.93 (1.28‐2.91)**	**.002**	75/279	54/299	**0.67 (0.46‐0.99)**	**.045**	54/298	75/280	**1.47 (1.00‐2.17)**	**.049**
Clinical stages																
I + II	78/863	19/94	**2.24 (1.30‐3.86)**	**.004**	62/753	35/204	**2.08 (1.34‐3.24)**	**.001**	61/438	36/519	**0.50 (0.32‐0.77)**	**.002**	36/518	61/439	**2.00 (1.30‐3.08)**	**.002**
III + IV	46/863	9/94	1.81 (0.86‐3.82)	.119	33/753	22/204	**2.47 (1.41‐4.34)**	**.002**	34/438	21/519	**0.52 (0.30‐0.91)**	**.021**	21/518	34/439	**1.92 (1.10‐3.37)**	**.022**

Abbreviations: AOR, adjusted odds ratio; CI, confidence interval.

^a^ Adjusted for age and gender, omitting the corresponding stratification factor.

### 3.3 Haplotype analysis

We also investigated the effects of *H19* haplotypes (Table 3), finding that the GGG (adjusted OR = 1.93, 95% CI = 1.41‐2.63) and AGG (adjusted OR = 1.41, 95% CI = 1.07‐1.85) haplotypes were significantly associated with an increased risk of hepatoblastoma compared to the GCA reference haplotype (order: rs2839698, rs3024270, rs217727).

**Table 3:** Frequency of inferred *H19* haplotypes based on their observed genotypes and their association with hepatoblastoma susceptibility

Haplotype^a^	Cases (n = 426)	Controls (n = 1914)	Crude OR (95% CI)	*P*	AOR (95% CI)^b^	*P* ^b^
GCA	105 (24.65)	622 (32.50)	1.00		1.00	
GCG	81 (19.01)	385 (20.11)	1.25 (0.91‐1.71)	.173	1.25 (0.91‐1.71)	.172
GGA	1 (0.23)	3 (0.16)	1.98 (0.20‐19.16)	.557	1.97 (0.20‐19.13)	.559
GGG	95 (22.30)	292 (15.26)	**1.93 (1.41‐2.63)**	**<.0001**	**1.93 (1.41‐2.63)**	**<.0001**
ACA	0 (0.00)	3 (0.16)	—	—	—	—
ACG	1 (0.23)	7 (0.37)	0.85 (0.10‐6.95)	.877	0.84 (0.10‐6.91)	.872
AGA	0 (0.00)	0 (0.00)	‐	—	—	—
AGG	143 (33.57)	602 (31.45)	**1.41 (1.07‐1.85)**	**.015**	**1.41 (1.07‐1.85)**	**.015**

Abbreviations: AOR, adjusted odds ratio; CI, confidence interval; OR, odds ratio.

^a^ Haplotype order: rs2839698, rs3024270, rs217727.

^b^ Obtained using logistic regression models adjusted for age and gender.

### 3.4 Effects of rs2839698, rs3024270 and rs217727 polymorphisms on gene expression

**Figure 1:**
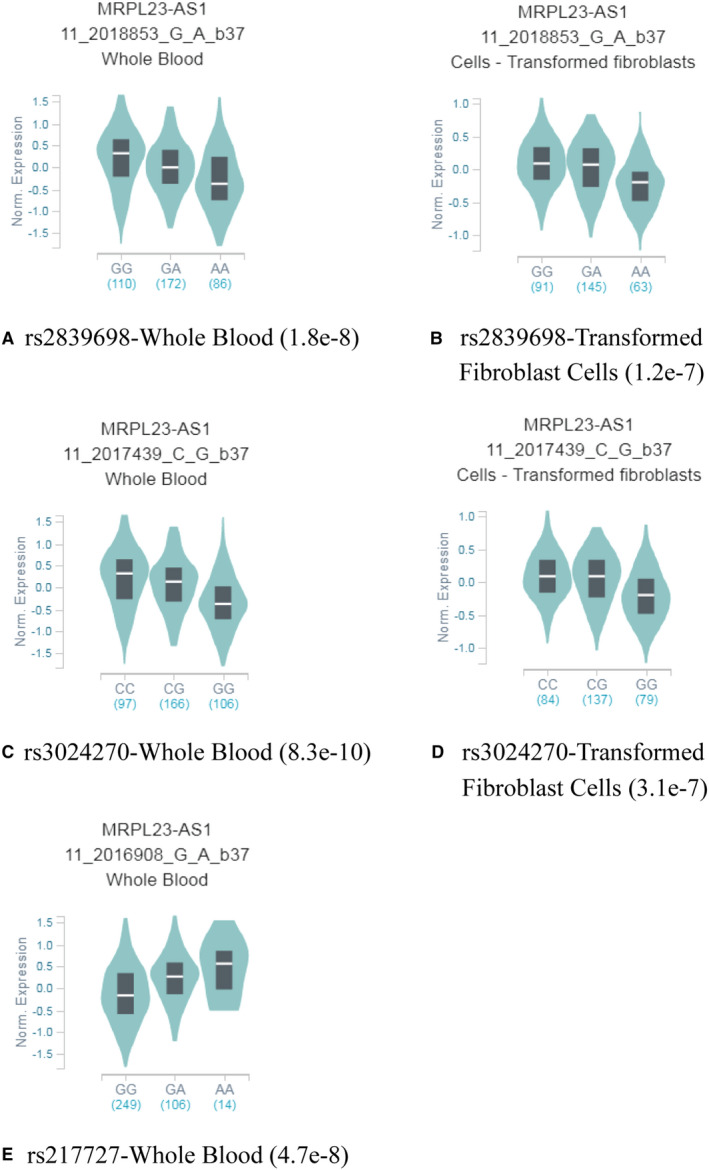
The rs2839698, rs3024270 and rs217727 genotypes correlated with MRPL23‐AS1 expression in whole blood (A, C and E) and transformed fibroblast cells (B, D and F) (data from GTEx Portal)

To explore the effect of rs2839698, rs3024270 and rs217727 polymorphisms on potential cancer‐related gene expression, we obtained eQTL evidence from the public GTEx (http://www.gtexportal.org/home/) database. We found that rs2839698 and rs3024270 were significantly correlated with decreased MRPL23‐AS1 expression (*P* < .001, Figure 1), whereas rs217727 was associated with increased MRPL23‐AS1 expression (*P* < .001, Figure 1).

## 4 DISCUSSION

LncRNAs play regulatory roles in a variety of biological processes, including tumorigenesis.^29^ Emerging evidence has suggested that lncRNA SNPs can affect the function and expression of cancer‐associated genes, leading to increased cancer susceptibility.^30^ In this case‐control study of 213 patients with hepatoblastoma and 958 healthy controls, we found that rs2839698, rs3024270 and rs217727 *H19* polymorphisms are significantly associated with hepatoblastoma susceptibility. To our knowledge, this is the first study to discover that *H19* polymorphisms are associated with hepatoblastoma susceptibility.

A variety of chromosomal abnormalities have been identified in patients with hepatoblastoma, such as the amplification or loss of DNA copies in chromosomes 5 and 11p15.5.^31^ Moreover, the inactivation of the *APC* gene, located on chromosome 5, has been observed in 67%‐89% of sporadic hepatoblastoma cases, while chromosome 11 aberrations may play an important role in hepatoblastoma pathogenesis in patients with BWS.^32^ Furthermore, Rumbajan et al^3^ found that abnormal DMR methylation occurs before hepatoblastoma development, suggesting that DMR methylation is related to hepatoblastoma occurrence. In this study, we found that lncRNA *H19* polymorphisms were significantly associated with hepatoblastoma risk.

H19 is an imprinted oncofetal gene that is located close to the *IGFII* gene locus,^33^ and its role in cancer has been widely studied. A meta‐analysis study showed that the rs2735971 A>G, rs2839698 C>T and rs3024270 G>C *H19* polymorphisms were associated with increased overall cancer risk.^34^ In addition, a population‐based case‐control study genotyped 177 patients with bladder cancer and 122 healthy controls for *H19* rs2839698, finding that the rs2839698 TC genotype significantly reduced the risk of bladder cancer compared to the rs2839698 TT genotype.^35^ Meanwhile, a genotype analysis of four *H19* SNPs (rs217727 C>T, rs2839698 C>T, rs3741216 A>T, rs3741219 T>C) in 500 patients with gastric cancer and 500 healthy controls showed that the rs217727T and rs2839698T allele carriers have increased risk of developing gastric cancer.^36^ Furthermore, the rs217727 (G>A) *H19* polymorphism has been associated with increased risk of osteosarcoma,^37^ whereas another study found that the polymorphism was also associated with increased risk of bladder cancer.^38^ However, in this study, we found that rs217727 (G>A) was associated with decreased risk of hepatoblastoma, with rs217727 AG genotype carriers having a lower risk of hepatoblastoma than GG genotype carriers. Taken together, these findings may enhance our understanding of the potential role of *H19* SNPs in cancer pathogenesis and suggest that *H19* SNPs may play an important role in the development of both adult and paediatric cancers.

LncRNA‐*H19* polymorphisms have also been associated with the risk and prognosis of patients with HCC, with haplotype analysis revealing that the GTC *H19* haplotype (rs2735971, rs2839698, rs3024270) significantly increased HCC risk.^32^ Moreover, in a study of *H19* polymorphisms and coronary artery disease (CAD) risk, haplotype analysis found that individuals with CGCC, TGAA and TAAA haplotypes had a higher CAD prevalence than the most common CGAC haplotype.^39^ In this study, we found that the GGG and AGG haplotypes were associated with a significantly higher risk of hepatoblastoma than the GCA haplotype (order: rs2839698, rs3024270, rs217727).

Previously, Li et al investigated the effects of rs2839698 C>T, rs3024270 G>C and rs217727 C>T in colorectal cancer in a Chinese population and their effects on the secondary structure of *H19* mRNA. All three SNPs dramatically altered the secondary structure of *H19* mRNA, while rs2839698 (C>T), which is located in the *H19* exon, may alter the activity and function of the H19 promoter by altering target miRNAs, such as hsa‐miR‐24‐1‐5p, hsa‐miR‐4486 and hsa‐miR‐566.^40^ MRPL23‐AS1 is a non‐coding cancer‐related gene located 5.9 kb downstream of *H19* rs2839701. Recent studies have shown that rs2839701 C>G inhibits transcriptional activity that may be associated with decreased MRPL23‐AS1 expression and raises the risk of oral squamous cell carcinoma.^41^ In this study, we found that *H19* rs2839698 and rs3024270 were correlated with decreased MRPL23‐AS1 expression, whereas rs217727 was associated with increased MRPL23‐AS1 expression, suggesting that rs2839698 and rs3024270 may inhibit transcriptional activity related to reduce MRPL23‐AS1 expression, whereas rs217727 may have the opposite effect.

This case‐control study has several limitations. Firstly, potential selection bias could have been introduced as both the cases and controls were enrolled in hospitals. Secondly, only three *H19* SNPs were investigated in this study, with others such as rs2735971 C>T having been excluded. Moreover, the biological function of these three SNPs remains unclear; thus, further functional studies are required. In addition, hepatoblastoma risk is caused by complex gene‐environment interactions; therefore, environmental factors should also be included in further risk assessment if possible.

In this study, we demonstrated the relationship between three *H19* SNPs and hepatoblastoma susceptibility: rs2839698 and rs3024270 increased hepatoblastoma risk, whereas rs217727 polymorphism decreased hepatoblastoma risk in a Chinese Han population. Further functional studies on these SNPs with larger populations and different ethnicities could verify the role of *H19* SNPs in hepatoblastoma.

## CONFLICTS OF INTEREST

All authors declare no conflict of interest.

## AUTHOR CONTRIBUTION

**Tianbao Tan:** Conceptualization (equal); Investigation (equal); Writing‐original draft (equal); Writing‐review & editing (equal). **Jiahao Li:** Conceptualization (equal); Investigation (equal); Writing‐original draft (equal); Writing‐review & editing (equal). **Yang Wen:** Data curation (supporting); Formal analysis (supporting); Investigation (supporting); Writing‐review & editing (supporting). **Yan Zou:** Conceptualization (supporting); Formal analysis (supporting); Methodology (supporting); Writing‐original draft (supporting); Writing‐review & editing (supporting). **Jiliang Yang:** Data curation (supporting); Formal analysis (supporting); Investigation (supporting); Writing‐review & editing (supporting). **Jing Pan:** Data curation (supporting); Formal analysis (supporting); Investigation (supporting); Writing‐review & editing (supporting). **Chao Hu:** Data curation (supporting); Formal analysis (supporting); Investigation (supporting); Writing‐review & editing (supporting). **Yuxiao Yao:** Data curation (supporting); Formal analysis (supporting); Investigation (supporting); Writing‐review & editing (supporting). **Jiao Zhang:** Data curation (supporting); Formal analysis (supporting); Investigation (supporting); Writing‐review & editing (supporting). **Yijuan Xin:** Data curation (supporting); Formal analysis (supporting); Investigation (supporting); Writing‐review & editing (supporting). **Suhong Li:** Conceptualization (supporting); Formal analysis (supporting); Methodology (supporting); Writing‐original draft (supporting); Writing‐review & editing (supporting). **Huimin Xia:** Conceptualization (supporting); Formal analysis (supporting); Methodology (supporting); Writing‐original draft (supporting); Writing‐review & editing (supporting). **Jing He:** Conceptualization (lead); Data curation (lead); Formal analysis (lead); Funding acquisition (lead); Investigation (lead); Methodology (lead); Project administration (lead); Resources (lead); Supervision (lead); Writing‐original draft (lead); Writing‐review & editing (lead). **Tianyou Yang:** Conceptualization (lead); Data curation (lead); Formal analysis (lead); Funding acquisition (lead); Investigation (lead); Methodology (lead); Project administration (lead); Resources (lead); Supervision (lead); Writing‐original draft (lead); Writing‐review & editing (lead).

## Supporting information

Table S1

## Data Availability

The data used to support the findings of this study are available from the corresponding author upon request.
