# Supplementary material for: Association between lncRNA‐H19 polymorphisms and hepatoblastoma risk in an ethic Chinese population
Source: J Cell Mol Med. 2020 Nov 24;25(2):742–50. doi: 10.1111/jcmm.16124 (PMC7812267; doi:10.1111/jcmm.16124)
Supplement: Supplementary file 1 — Table S1 [file JCMM-25-742-s001.doc]

| **Supplemental Table 1**. Frequency distribution of selected variables in hepatoblastoma patients and controls | | | | | |
| --- | --- | --- | --- | --- | --- |
| Variables | Cases (n=213) | | Controls (n=958) | | *P^a^* |
|  | No. | % | No. | % |  |
| Age range, month | 0.23-149.97 | | 0.004-156.00 | | 0.105 |
| Mean ± SD | 23.62 ± 24.36 | | 23.75 ± 18.30 | |  |
| <17 | 114 | 53.52 | 454 | 47.39 |  |
| ≥17 | 99 | 46.48 | 504 | 52.61 |  |
| Gender |  |  |  |  | 0.973 |
| Female | 84 | 39.44 | 379 | 39.56 |  |
| Male | 129 | 60.56 | 579 | 60.44 |  |
| Clinical stages |  |  |  |  |  |
| I | 42 | 19.72 |  |  |  |
| II | 55 | 25.82 |  |  |  |
| III | 40 | 18.78 |  |  |  |
| IV | 15 | 7.04 |  |  |  |
| NA | 61 | 28.64 |  |  |  |
| SD, standard deviation, NA, not available.  ^a^ Two-sided χ^2^ test for distributions between hepatoblastoma patients and cancer-free controls. | | | | | |
